# Alterations of the miR-126-3p/POU2AF1/Spi-B Axis and JCPyV Reactivation in Multiple Sclerosis Patients Receiving Natalizumab

**DOI:** 10.3389/fneur.2022.819911

**Published:** 2022-03-11

**Authors:** Roberta Mancuso, Simone Agostini, Ambra Hernis, Domenico Caputo, Daniela Galimberti, Elio Scarpini, Mario Clerici

**Affiliations:** ^1^IRCCS Fondazione Don Carlo Gnocchi, Milan, Italy; ^2^Department of Biomedical, Surgical and Dental Sciences, University of Milan, Milan, Italy; ^3^Fondazione Cà Granda, IRCCS Ospedale Maggiore Policlinico, Milan, Italy; ^4^Department of Pathophysiology and Transplantation, University of Milan, Milan, Italy

**Keywords:** circulating microRNA (miRNA), natalizumab (Tysabri®), JCPyV, multiple sclerosis, rehabilitation, progressive multifocal leukoencephalopathy (PML)

## Abstract

Natalizumab (NTZ) can reactivate human polyomavirus John Cunningham polyomavirus (JCPyV) latent infection and lead to progressive multifocal leukoencephalopathy (PML). NTZ modulates the expression of microRNA-126-3p (miR-126-3p) and its target genes, *Spi-B, POU2AF1*, and vascular cell adhesion molecule-1 (VCAM-1); Spi-B protein binds the JCPyV regulatory region, initiating early gene transcription. This paper is aimed to evaluate the miR-126-3p and soluble (s)VCAM-1 concentration, *Spi-B*/*POU2AF1* gene expression, and JCPyV activity in patients with multiple sclerosis (MS) before and during 2-years NTZ. Serum miR-126-3p and sVCAM-1 concentration was measured before NTZ and after 1, 12, and 24 months of treatment in 22 MS subjects, 1 patient who developed PML, and 29 healthy controls (HCs). The *Spi-B* and *POU2AF1* expression in blood was analyzed at baseline and at month 24 in 13 patients with MS; results were clusterized based on JCPyV activity. miR-126-3p was significantly downregulated in MS before and during NTZ but was greatly increased in the PML patient. sVCAM-1 concentration was comparable in MS and HCs, and was reduced by NTZ in MS and PML. *Spi-B/POU2AF1* expression was significantly increased in MS at baseline and was upregulated by NTZ, particularly in JCPyV-infected patients in whom JCPyV reactivation was detected. Taken together, the results suggest that the modulation of the miR-126-3p/*POU2AF1/Spi-B* axis associates with JCPyV activity in NTZ-treated patients with MS.

## Introduction

Epigenetic mechanisms regulating gene expression, such as microRNA (miRNAs), are critical factors in complex human neurological diseases. miRNAs are highly conserved short non-coding RNAs that bind to complementary sequences on 3' untranslated region (3'UTR) of mRNAs resulting in translational repression or target degradation (1). Since miRNAs can be detected in biological fluids, they are expected to become biological biomarkers for the diagnosis, prognosis, and response to treatment in a number of diseases, such as multiple sclerosis (MS).

Multiple sclerosis is the most common demyelinating disorder of the central nervous system (CNS) and still only has a partially understood pathogenesis that includes environmental and genetic factors (2). Biological agents, such as monoclonal antibodies (mAbs), are currently considered as therapeutic options for MS (3); among such mAbs natalizumab (NTZ) (4) is a humanized anti-α4 integrin antibody. The use of NTZ results in clinical benefits and is associated with diverse biological effects, such as an increase in the number of peripheral immune cells, as a consequence of its ability to reduce the expression of very late activating antigen-4 (VLA-4) expression on leukocytes (5) and vascular cell adhesion molecule-1 (VCAM-1) on vascular endothelial cells (ECs) (4). In addition, NTZ results in a decrease of soluble (s)VCAM-1 and of intrathecal immune cells (6), and in the upregulation of the transcription regulators, POU domain class 2 associating factor 1 (POU2AF1) and Spi-B in lymphocytes (7).

Natalizumab can very rarely result in progressive multifocal leukoencephalopathy (PML), an extremely severe adverse event (8), which is caused by reactivation in the central nervous system (CNS) of human polyomavirus John Cunningham polyomavirus (JCPyV), a ubiquitous virus that latently infects the majority of humans. The mechanism of development of PML is not clear, and probably depends on the interaction between a number of factors (9); one of the most important is the altered immune surveillance to virus in CNS, but others unknown aspect may contribute to the development of this condition. JCPyV reactivation is driven by host nuclear transcription factors (10) binding to the specific sites of the JCPyV genome, (hypervariable non-coding control region, or NCCR), result in the activation of the viral lytic phase and the production of viral progeny (9). NCCR rearrangements with acquisition of multiple binding sites for transcription factors play a fundamental role in JCPyV neurovirulence, as this leads to the increased replication rate observed in glial cells (11).

The influence of different therapeutic agents used in patients with MS on the expression of circulatory miRNA has been previously investigated (12). NTZ, in particular, modifies miRNA expression profiles in patients with MS (13), reducing the expression of miR-126-3p in peripheral lymphocytes (7) and increasing that of the two of the targets of this miRNA: the transcription factors POU2AF1 and Spi-B (14). No data are nevertheless available on the possible effect of NTZ on miR-126-3p in serum. We performed a longitudinal analysis of serum miR-126-3p concentration in patients with MS treated with NTZ over a 24-months period. The serum concentration of sVCAM-1, *Spi-B*, and *POU2AF1* gene expression in lymphocytes was analyzed and evaluated in relation with NTZ-associated changes in JCPyV activity.

## Materials and Methods

### Patients and Controls

The study enrolled 22 patients with a diagnosis of relapsing remitting MS (RRMS) according to the 2005 revised McDonald criteria (15), who were taking part in a rehabilitation program and were followed at the ‘Don Gnocchi Foundation,' Milan, Italy. NTZ eligibility followed the guidelines of the Italian Medicines Agency (AIFA). The NTZ washout period was 3 months for patients previously using immune-modifying drugs, and 6 months for patients undergoing immunosuppressive therapy (mandatory according to AIFA guidelines).

All patients received intravenous NTZ (standard interval dosing, every 4 weeks) for a median period of 24 months (only 1 patient was treated for 20 months); 19/22 patients with MS continued NTZ therapy after the initial cycle of 24 infusions (mean 43 infusions and range 33–74) and are still followed at the Don Gnocchi Foundation. Clinical monitoring of these patients shows that no one has so far developed PML. At the initiation of therapy, mean age was 38.5 ± 9.6 years (SD), and mean expanded disability status scale (EDSS) score was 4.5 (interquartile range [IQR]: 3.5–5.0).

The control group included 29 sex- and age-matched healthy control (HC) subjects who donate blood at the same institution, with a mean age of 43.3 ± 10.1 years. Demographic and clinical data of patients with MS and HCs are presented in Table 1.

**Table 1:** The demographic and clinical data of the relapsing remitting multiple sclerosis (RRMS) subjects and healthy controls (HCs) enrolled for this study.

	**RRMS**	**HC**
- Number	22	29
- Sex (males/females)	4/18	10/19^**^
- Age (mean ± SD, years)	38.5 ± 9.6	43.3 ± 10.1∧
- Age at onset (mean ± SD, years)	26.8 ± 7.2	
- Disease duration (mean ± SD, years)	11.5 ± 6.8	
- EDSS at baseline (median, IQR)	4.5 (3.5–5.0)	
- EDSS after 2 years of treatment (median, IQR)	4.5 (2.5–5.0)^*^	
- Patients with MRI activity in the first year of treatment	8 (36%)	
- Patients with MRI activity in the second year of treatment	2 (9%)	
- Patients with freedom from disease activity during treatment	12 (54%)	
- Patients with previous DMT (%)	15 (68%)	
- Patients with previous IST (%)	6 (27%)	

*Data are expressed as means ± SD or median and interquartile range (IQR); EDSS, Expanded Disability Status Scale; MRI, magnetic resonance imaging; DMT, disease modifying therapies, i.e., beta-interferons or glatiramer acetate; IST, immunosuppressive therapies, i.e. azathioprine or mitoxantrone*.

* *p = n.s. vs. baseline (Wilcoxon's test)*;

** *p = n.s. vs. RRMS (Fisher's exact test); ∧p = n.s. vs. RRMS (Student's t-test)*.

The study was authorized by the Ethical Committee of the Don Gnocchi Foundation and was performed in accordance with the Declaration of Helsinki. An informed consent was signed by all the participants.

Peripheral blood samples were collected before the first infusion (*baseline*) and 1, 12, and 24 months after NTZ initiation; peripheral blood was collected once for HC subjects. Blood specimens were collected in the morning from fasting participants; for patients with MS, the sampling was performed just before the NTZ infusion. JCPyV serostatus and the presence of viral DNA in blood and urine in these patients has been previously described (16).

One additional patient (female, 38 years old) who developed a severe PML after 2 years of NTZ was also included in the study. This patient was followed at the Ospedale Maggiore Policlinico, (Milan) and developed the disease after 21 infusions of NTZ with clinical stability for about 2 years, showing very high JCPyV viral load in cerebrospinal fluid, acute language disorder, and diffuse signal alteration in the deep white matter of the brain evidenced by magnetic resonance imaging (MRI) (17). Then, 2 months after plasma exchange to remove drug, the clinical stability was achieved; shortly thereafter, though, she developed a severe immune reconstitution inflammatory syndrome (IRIS) with significant worsening of clinical status (hemiparesis and decreases of consciousness) and the progression of mass lesions at MRI: a high dose of steroid treatment lead to a partial clinical recovery (17). For this patient, we analyzed serum samples collected at the time of PML development and 1 year later, when a partial recovery was observed.

### Serum miRNAs Extraction and Reverse Transcription

Sample handling procedures were the same for MS and HC subjects. In particular, sera from 22 patients with RRMS, 29 HCs, and 1 PML individual were obtained by centrifugation (1,500 *g* ×10min, RT) and stored at −80°C until use. The absence of hemolysis in sera was evaluated by visual inspection and by the spectrophotometric measurement of absorbance of hemoglobin at 414 nm (18). The miRNAs were extracted from 200 μl serum using the miRNeasy serum/plasma Kit (Qiagen GmbH, Hilden, Germany) by robotic workstation (Qiacube, Qiagen) according to the specific manufacturer's instructions and kept at −80°C until cDNA synthesis. The miRNA content was quantified with a Qubit microRNA assay kit (Thermo Fisher Scientific, Foster City, CA, USA) with a Qubit 3.0 Fluorometer (Thermo Fisher Scientific), according to manufacturer's recommendations. A reverse-transcription reaction was performed with Universal cDNA synthesis kit II (Qiagen), using 5 ng of miRNA, with the following protocol: 60 min at 42°C, followed by the heat-inactivation of reverse transcriptase for 5 min at 95°C. To avoid variation due to sample differences and handling, all the variables involved in the procedure were kept consistent throughout the study.

### Quantification of Serum miR-126-3p by Digital Droplet PCR (ddPCR)

MicroRNA-126-3p was quantified by digital droplet PCR (ddPCR) using specific miRCURY LNA miRNA assays (Qiagen). ddPCR workflow and data analyses were performed according to the manufacturer's instructions. Briefly, cDNA (10x) was partitioned into ~20,000 water-in-oil droplets. A no template control and a negative control for each reverse transcription reaction were included in every assay to check for no specific amplification. The droplets were transferred into a plate, followed by end-point amplification. Afterward, the plate was loaded into the Droplet Reader. Droplet reading was done with a QX200 droplet reader (Bio-Rad, Hercules, CA, USA) that quantifies the positive and negative droplets, depending on the presence or absence of template. The absolute number of copies was quantified using the Quantasoft software analysis, version 1.7.4.0917 (Bio-Rad).

### Spi-B/POU2AF1 Gene Expression Analysis in Lymphocytes by Quantitative PCR (qPCR)

The *Spi-B/POU2AF1* gene expression analysis in peripheral blood mononuclear cells (PBMCs) could be performed at baseline and after 24 months of therapy in 13 patients with RRMS and 10 age- and sex-matched HCs. PBMCs were isolated from peripheral blood by density gradient centrifugation *(*Lympholyte Separation Medium, Cedarlane, Hornby, Ontario, CA, USA) and stored at −80°C until use.

Total RNA was extracted from PBMCs (RNAeasy Mini extraction kit, Qiagen), quantified and reverse-transcribed to cDNA, as previously described (19). RNA quality and purity were assessed by the analysis of spectrophotometric of 260/280 (≥2.0) and 260/230 ratio (range of 2.0–2.2). Pre-designed and validated assays (Thermo Fisher Scientific) were used for relative quantification of the target genes (*Spi-B* and *POU2AF1*) and for two previously selected housekeeping genes (*GAPDH, YWHAZ*). Quantitative PCR (qPCR) experiments were performed on CFX Touch real-time PCR (Bio-Rad) in triplicate, including the positive and non-template controls. All procedures were carried out under stringent conditions to avoid contamination. A relative expression analysis was performed by qBase+ software [Version: 3.0, Biogazelle], using a comparative cycle threshold method (20). The change in gene expression fold compared with baseline (delta fold) was calculated for each subject.

### sVCAM-1 Quantification in Serum

Soluble vascular cell adhesion molecule-1 concentration was determined by using an enzyme linked immunosorbent assay (sVCAM-1 ELISA KIT, IBL, Hamburg, German), according the manufacturer's instructions. Serum samples were diluted 1:50 in Assay Buffer and tested in duplicate. Optical densities were determined for each well of plate (Sunrise, Tecan, Mannedorf, Switzerland), calculating the concentration of samples from a standard curve. The limit of detection was 0.6 ng/ml.

### Defining Patients in Relationship With JCPyV Infection and Activity

Patients with MS were divided into three groups, based on the presence/absence of JCPyV infection and reactivation as follows: ***uninfected:*** 3 patients that were seronegative for JCPyV antibodies and with undetectable JCPyV DNA in blood and urine at baseline and during 24-month NTZ treatment. ***JCPyV latency:*** 3 patients that were seropositive for JCPyV antibodies and in whom viral DNA was undetectable in blood and urine (viral latency during treatment). ***JCPyV activity:*** 7 patients who were seropositive for JCPyV antibodies and in whom JCPyV DNA was detected in urine and/or blood (asymptomatic viral reactivation). JCPyV serostatus was determined with ELISA test performed by a centralized service supported (STRATIFY project, Biogen Idec) (21).

### Data Analysis and Statistics

Parametric data are summarized as mean ± SD, comparisons among groups were analyzed by ANOVA test and Student's *t*-test, when appropriate. Non-parametric data were reported as median and IQR (IQR: 25th and 75th percentile), and comparisons were analyzed by Kruskal–Wallis and Mann–Whitney *U*-test, or with Wilcoxon signed-rank test for paired data, as appropriate. Pre- vs. post-treatment variables differences (delta) were calculated for each subject and compared with non-parametric tests (Kruskal–Wallis, and Mann–Whitney tests). Correlations were analyzed using Spearman's correlation coefficient. Receiver operating characteristics analysis (ROC) and area under curve (AUC) were used to evaluate the potential of miRNA as biomarker.

## Results

### Serum miR-126-3p Serum Concentration in NTZ-Treated Patients With RRMS

**Figure 1:**
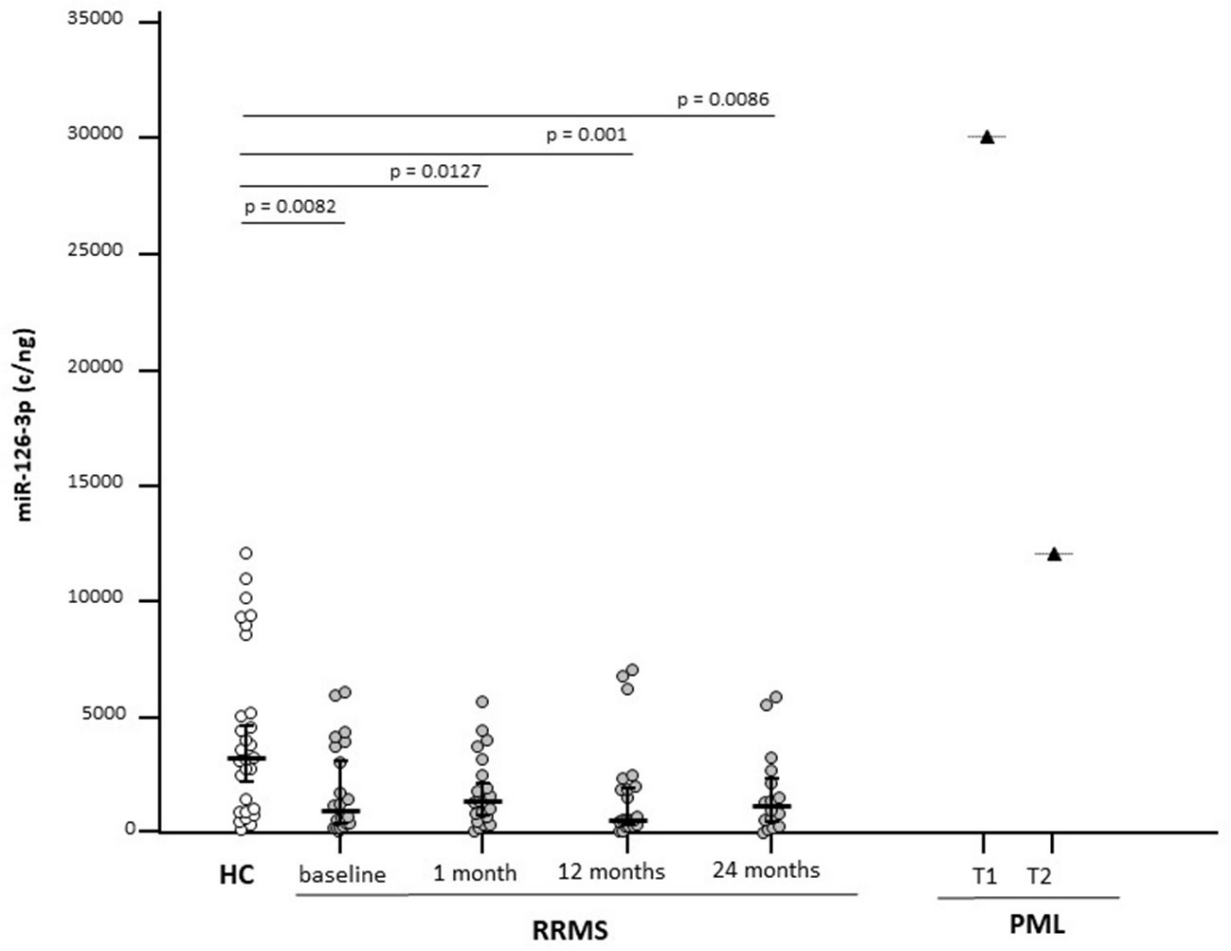
Serum microRNA-126-3p (miR-126-3p) concentrations in patients with a diagnosis of relapsing-remitting multiple sclerosis (RRMS) (*N* = 22) or progressive multifocal leukoencephalopathy (PML) (*N* = 1) and in age-and sex-matched healthy controls (HCs) (*N* = 29). Results before (baseline) and at 3 different time points are initiation of natalizumab (NTZ) therapy are shown. For the patients with PML, time at PML diagnosis (T1) and 12 months after PML diagnosis (T2) are shown. miR-126-3p expression (copies/ng) is presented as median and interquartile range (IQR). Statistical significances are indicated.

MicroRNA-126-3p serum concentration was significantly decreased in RRMS compared with healthy controls (*p* = 0.0082) at baseline (Figure 1) and remained significantly decreased compared with HC at every time point after NTZ initiation, but without significant difference among the time points. Thus, a transient increase from baseline was observed 1 and 24 months after initiation of NTZ, whereas the value at 12 months was similar to that of baseline (Table 2).

**Table 2:** Serum miR-126-3p concentration (copies/ng) in RRMS, PML, and HC subjects.

	**miR-126-3p (c/ng)**						
	**RRMS (*n =* 22)**	**RRMSJCPyV uninfected(*n =* 5)**	**RRMS** **JCPyV** **infected** **(*n =* 17)**	**RRMSJCPyV latency(*n =* 6)**	**MS** **JCPyV activity (*n =* 11)**	**PML(*n =* 1)**	**HC (*n =* 29)**
**Baseline**	941.5^**a**^ (400.0; 3,740.0)	540.0(415.0; 2,191.5)	1,175.0 (254.9; 3,729.0)	2,150.0(560.0; 595.0)	708.0 (242.5; 3,235.0)	na	3,267.0^**a**^ (995.0; 6,050.0)
**1 month**	1,490 (692.0; 2,517.0)	1,016.0(342.0; 1,960.0)	1,608.5 (850.0; 3,455.0)	2,544.0(1,380.0; 4,417.0)	1,320.0 (800.0; 1,792.0)	na	
**Delta(c/ng)**	−25 (−903; 800)	−100(−1,043; 156)	75 (−986; 942)	−120(−1,152; 140)	321 (−820; 1,084)		
**12 months**	562.0 (367.0; 2,017.0)	483.5(300.0; 1,900.0)	637.0 (423.0; 2,183.5)	549.0(300.0; 717.0)	2,017.0 (465.0; 5,705.0)	30,083.0	
**Delta(c/ng)**	117 (−961; 1,002)	– 117^b^(−402; 1564)	178 (−1,399; 830)	−1,758 ^b, c^(−4,050; −260)	530^c^ (117; 1,309)		
**24 months**	1,360.0 (550.0; 2,667.0)	1,533.0(1,139.0; 8,946.5)	950.0 (482.5; 2,256.7)	905.0(570.0; 2,095.0)	1,340.0 (449.0; 2,256.7)	12,083.0	
**Delta(c/ng)**	−176 (−945; 591)	1,133(100; 1,145)	−340 (−1,549; −42)	−1,545(−3,921; −165)	−108 (−785; 315)		

*Expression values obtained at baseline and at three different time points after initiation of Natalizumab treatment are shown; for PML patients, time at PML diagnosis and after 12 months from the diagnosis. Variation of mir-126-3p expression at each time point from baseline (delta) are reported. Median and interquartile range (IQR) are indicated. Wilcoxon or Mann-Whitney tests were used to combreake respectively paired or unpaired data. RRMS, relapsing remitting multiple sclerosis; PML, Progressive Multifocal Leukoencephalitis; HC, healthy controls; JCPyV, JC polyomavirus; delta, difference of the values obtained at different time point to baseline; na, not available*;

a *p = 0.0082 (Mann–Whitney test)*;

b *p = 0.044 (Mann–Whitney test)*;

c *p = 0.0126 (Mann–Whitney test)*.

**Figure 2:**
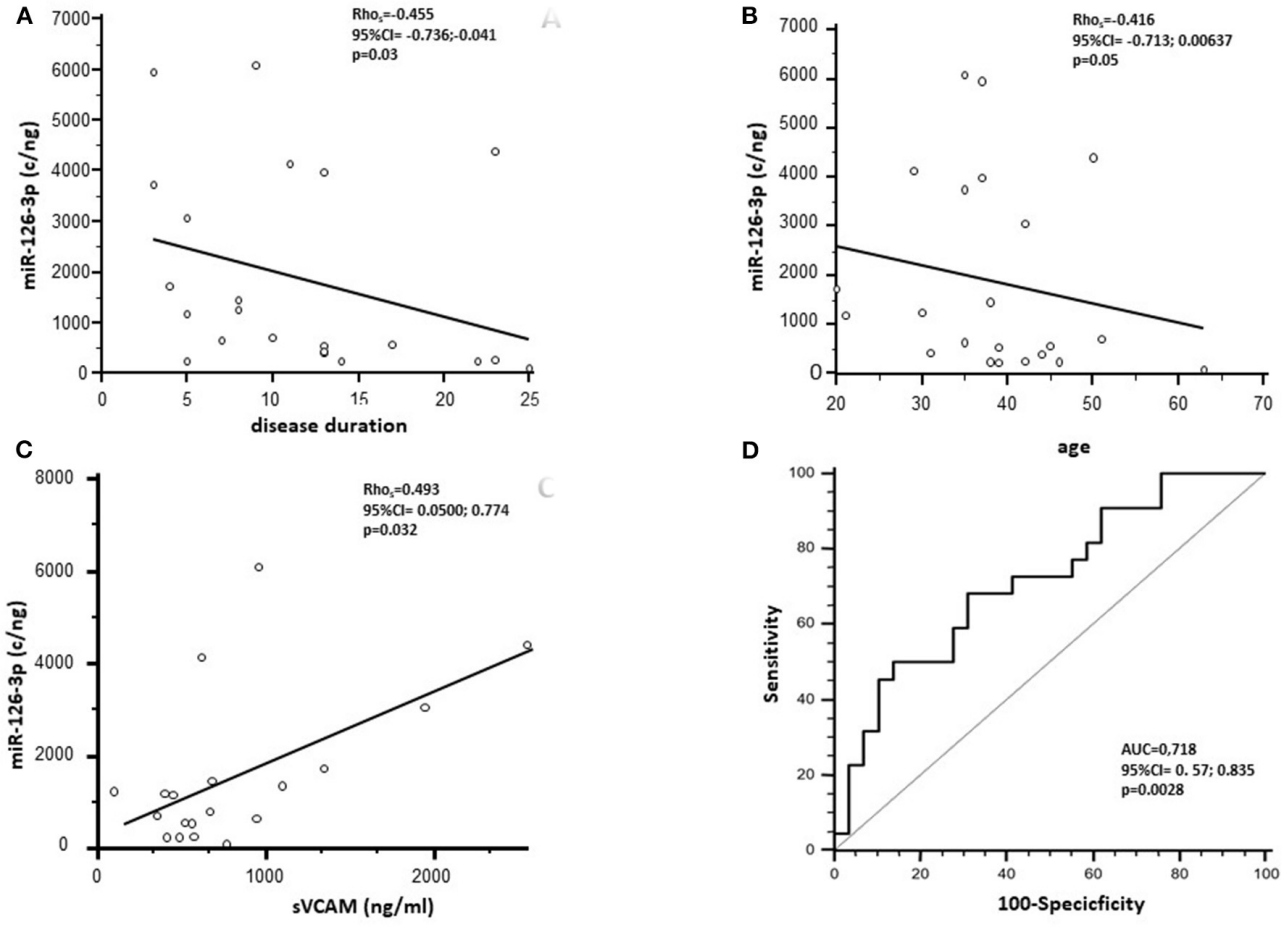
Spearman's correlation analysis between the miR-126-3p serum concentration (copies/ng) of RRMS subjects before NTZ treatment and disease duration **(A)** and age **(B)**. **(C)** Represents Spearman's correlation between miR-126-3p serum concentration (copies/ng) and soluble (s)VCAM-1 in RRMS subjects. **(D)** Presents receiver operating characteristic curve (ROC) for the prediction of RRMS based on miR-126-3p serum concentration. AUC, area under the curve.

At baseline, miR-126-3p serum concentration was negatively correlated with disease duration (*p* = 0.03) and with age (*p* = 0.05) (Figures 2A,B). No significant correlations were observed between this parameter and either gender, age at disease onset, or EDSS. Analysis with the AUC of ROC showed that miR-126-3p serum concentration can discriminate between MS and HC (*p* = 0.0028), with a 68.2% sensitivity and a 67.9% specificity (Figure 2D).

Analyses performed on the patient who developed PML showed a >9-fold increase of miR-126-3p serum concentration at the time of PML diagnosis compared with the values seen in HC; interestingly, miR-126-3p serum concentration was significantly decreased when a clinical partial recovery was observed, although it remained well above the concentration observed in either HC or RRMS (Figure 1).

### Serum sVCAM-1 Concentration in NTZ-Treated Patients With RRMS

**Figure 3:**
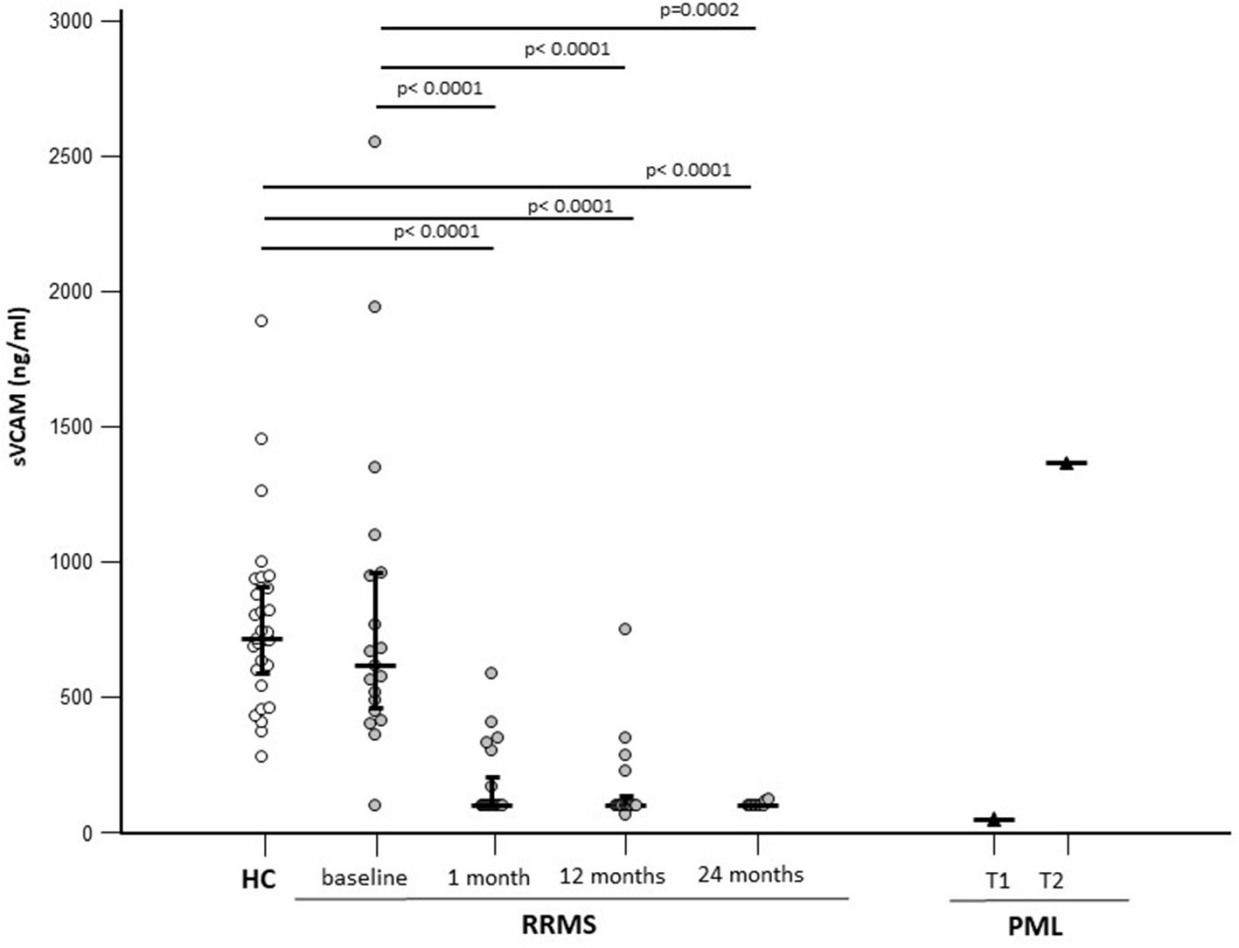
Serum sVCAM-1 concentrations in patients with a diagnosis of RRMS (*N* = 22) or PML (*N* = 1) and in age-and sex-matched HCs (*N* = 29). Results before (baseline) and at 3 different time points are initiation of NTZ therapy are shown. For the patients with PML time at PML diagnosis (T1) and 12 months after PML diagnosis (T2) are shown. The expression of miR-126-3p (copies/ng) is presented as median and IQR. Statistical significances are indicated.

As VCAM*-*1 is one of the targets of miR-126*-*3p, the concentration of sVCAM-1 was measured in the sera of patients with RRMS and HCs to verify the possible effect of NTZ on the expression of this protein and to study its relation with the expression of circulatory miR-126-3p. The sVCAM-1 concentration was comparable in RRMS (618.15; 439.83–995.52 ng/ml) and HCs (median: 717.35; IQR 584.68–908.56 ng/ml) at baseline and was significantly reduced by NTZ at every time point (median: 100 ng/ml for all time points; *p* < 0.005 compared with baseline) (Figure 3).

In the patient who developed PML, serum sVCAM-1 concentration was comparable to that seen in NTZ-treated patients (100 ng/ml) at the time of diagnosis; at the time of partial recovery, though, serum sVCAM-1 concentration was greatly increased (1,365 ng/ml) (Figure 3).

A significant positive correlation was observed between sVCAM-1 serum baseline concentration and miR-126-3p in patients with RRMS alone (*p* = 0.032) (Figure 2C).

There were no gender- or age-based differences observed in sVCAM concentration in either patients with MS or HCs.

### Baseline *Spi-B* and *POU2AF1* Gene Expression in PBMCs of Patients With RRMS

MicroRNA-126-3p regulates POU2AF1 expression, this results in the modulation of *Spi-B* expression; we measured the gene expression levels of these two transcription factors in PBMCs of a subgroup of 13 patients with RRMS, 10 of whom were JCPyV-infected and three were not JCPyV-infected (as shown in Material and methods). Results showed that both *Spi-B* and *POU2AF1* expression was significantly increased at baseline in patients with RRMS compared with HCs (*p* = 0.0043 and *p* < 0.001, respectively) (Table 3). The gene expression of *Spi-B* and *POU2AF1* in PBMCs was not different in male and female patients.

**Table 3:** The gene expression of *Spi-B* and *POU2AF1* in PBMCs form natalizumab (NTZ) treated patients with RRMS.

	**Baseline**	**After NTZ**	***p-*value**	**Δ Fold**
***Spi*****–*****B gene expression in PBMCs;*** **median fold, (IQR)**				
Total RRMS (nr = 13)	2.33^a^ (1.59–4.70)	5.30 (3.23–8.33)	0.0034	2.01 (104503)
JCPyV uninfected RRMS (nr = 3)	5.85^b^ (3.48–6.36)	4.70 (4.67–6.27)	ns	0.27 (−0.82–1.57)
JCPyV infected RRMS (nr = 10)	2.04^b^ (1.28–3.72)	5.48 (3.07–9.34)	0.0059	4.50 (1.59–5.68)
JCPyV latency (nr = 3)	1.69 (1.03–3.82)	3.28 (2.88–7.82)	ns	1.87 (1.66–4.07)
JCPyV activity (nr = 7)	2.31 (1.41–3.37)	5.66 (3.63–13.03)	0.047	4.62 (2.07–10.71)
HCs	0.98^a^ (0–80–1.26)			
*POU2AF1 gene expression in PBMCs*; median fold, (IQR)				
Total RRMS (*n =* 13)	3.49^c^ (2.65–4.75)	7.21 (5.23–12.90)	ns	4.07(0,81– 9,76)
Uninfected RRMS (*n =* 3)	3.89 (3.68–13.05)	5.82 (5.58–7.29)	ns	1.61 (−5.85–2.06)^d^
JCPyV infected RRMS (*n =* 10)	3.23 (2.59–4.69)	8.02 (5.10–13.22)	ns	4.36 (1.55–10.63)
JCPyV latency (*n =* 3)	4.69 (3.18–15.83)	4.22 (3.51–7.68)	ns	−1.42 (−8.39 −0.81)^e^
JCPyV activity (*n =* 7)	3.14 (1.29–3.45)	12.79 (5.76–14.03)	0.0156	9.47 (4.33–10.76)^d, e^
HC	0.805^c^ (0.687–1.628)			

*Expression fold, relative to HCs, are expressed as median IQR. Δ fold = variation of gene expression fold from baseline. Ns, not significant. PBMCs, peripheral blood mononuclear cells; JCPyV, JC polyomavirus. Significance of Mann–Whitney test or Wilcoxon test statistics in the difference between unpaired groups or paired groups are reported*;

a *p = 0.0043*;

b *p = 0.042*;

c *p < 0.001*;

d, e *p = 0.0167*.

### Analysis of Results in Relation to JCPyV Activity

To evaluate possible correlations between the NTZ-associated modulation of *Spi-B* and *POU2AF1* expression and miR-126-3p and sVCAM-1 serum concentration, with the presence/lack of JCPyV reactivation, we divided patients into “*JCPyV uninfected*” and “*JCPyV infected*” individuals; *JCPyV infected* individuals were further divided into patients with “*JCPyV latency”* or with “*JCPyV activity.”* For each MS subject, the virological characterization is summarized in Supplementary Figure S1.

At month 12, miR-126-3p serum concentration was decreased in *JCPvV latency* patients (variation from baseline, delta median: −1,758 c/ng), but was significantly increased in those classified as “*JCPyV activity* (delta: 530 c/ng; *p* = 0.0126). These differences were maintained at month 24: *JCPvV latency*, delta: −1,545; “*JCPyV activity*: delta: −108). In contrast with these results, no correlation could be detected at any time point between JCPyV status and sVCAM-1 serum concentration.

**Figure 4:**
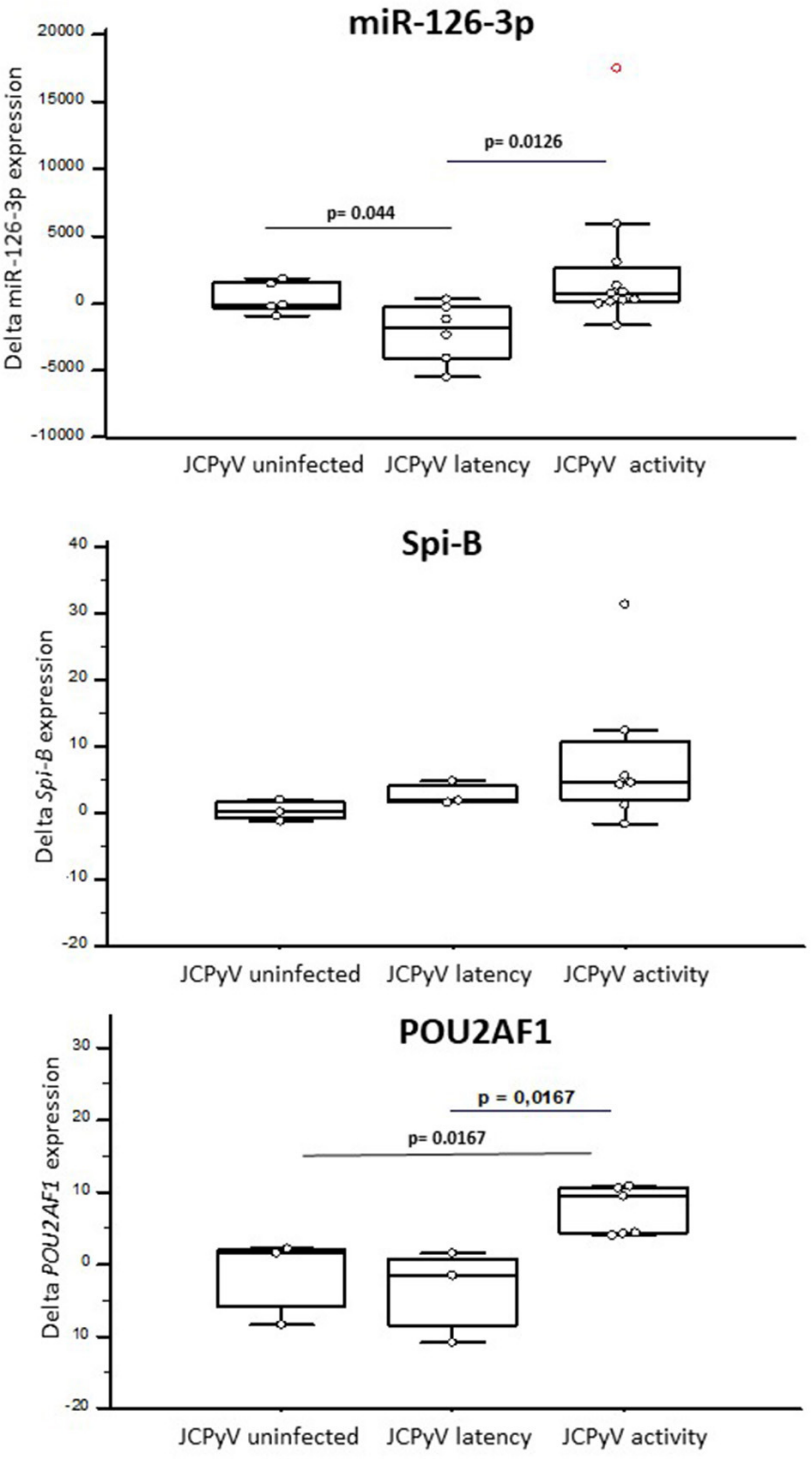
The expression of miR-126-3p **(A)**, *POU2AF1* **(B)**, and *Spi-B* **(C)** in RRMS patients treated with NTZ, clusterized based on the lack of JCPyV infection (JCPyV uninfected) (*N* = 3) or on its presence in a latent (latency) (*N* = 3) or replicating (activity) (*N* = 7) form. Increases or decreases (delta) compared with baseline after NTZ therapy are shown as box and whiskers plot, where the box indicates the median and the first and third quartiles; the whiskers extend from the lowest to the highest values of the dataset. Statistical significance (Mann–Whitney test) is indicated.

Natalizumab increased both *Spi-B* (Δfold, median: 4.50) and *POU2AF1* (Δfold: 4.36) expression in “*JCPyV infected*” patients alone. Notably, patients with “*JCPyV activity”* (*Spi-B*, Δfold: 4.62; *POU2AF1*, Δfold: 9.47) was clearly different to those with “*JCPyV latency*,” in whom *POU2AF1* significantly decreased (Δfold−1.42; *p* = 0.0167) and *Spi-B* increased (Δfold: 1.87). The variation of expression (delta) from baseline for miR126-3p, *POU2AF1*, and *Spi-B* is shown in Figure 4.

The expression of miR-126-3p, *Spi-B*, and *POU2AF1* was not significantly different when patients with MS in whom disease activity during therapy was or was not detected were compared.

## Discussion

John Cunningham polyomavirus serostatus, antibodies index, previous immunosuppressive therapies, and duration of NTZ treatment are currently used for PML risk stratification in patients with MS undergoing NTZ (22); these biomarkers, nevertheless, have a very limited ability to predict the risk of developing PML. Herein, we present results obtained by monitoring the different host factors that could be critical elements for the reactivation of JCPyV in patients with RRMS who had been treated with NTZ for 2 years: these patients were already characterized from a virological point of view (16).

Although the risk of PML increases after 24 months of treatment (23), the ongoing clinical monitoring of our patients who have been receiving NTZ for prolonged periods of time shows that none of them has developed PML.

We analyzed miR-126-3p concentration using the digital droplet PCR, an extremely accurate methodology that provides directly the absolute number of copies of miRNA in serum, as well as the expression of *POU2AF1* and *Spi-B*, two genes regulated by this miRNA. Moreover, we evaluated the effect of NTZ on another putative target of miR-126-3p: sVCAM-1, a protein involved in leukocyte adhesion to ECs, a mechanism that is inhibited by NTZ (5, 24). Results showed that, as compared with healthy controls, at baseline miR-126-3p serum concentration was significantly reduced and *POU2AF1/Spi-B* PMBCs expression was significantly increased in RRMS, whereas sVCAM-1 serum concentration was similar in the two groups. NTZ treatment only marginally changed miR-126-3p serum concentration, but resulted in the upregulation of *POU2AF1* and *Spi-B* expression and in a significant decrease of sVCAM-1 serum concentration.

To the best of our knowledge, this is the first study to examine miR-126-3p serum concentration in RRMS. Results showing that this miRNA is significantly downregulated in patients in accordance with data obtained in other autoimmune diseases, such as systemic lupus erythematosus (SLE) and type 2 diabetes mellitus (25), which are characterized by chronic inflammatory response. These findings thus indicate that a reduction of miR-126-3p concentration is a common feature in inflammatory diseases.

MicroRNA-126-3p expression has been previously described to be reduced in the whole blood of MS subjects (26); other results showed this molecule to be augmented instead in the lymphocytes of patients with RRMS (27). Several factors may account for this discrepancy, including the methods used for the analysis (qPCRvs ddPCR) and for results normalization. Notably, our data stem from ddPCR analyses and present miR-126-3p absolute concentration, while previous studies used qPCR and relative expression after the normalization of raw data (28).

Different cell types and a variety of mechanisms contribute to the generation of circulating miRNAs. miR-126, in particular, can be secreted inside extracellular vesicles (29), which are mostly originated from vascular ECs (30) and from CD34+ hematopoietic precursors (31), but can also derive from broken, apoptotic, or necrotic cells (32). As NTZ is known to increase the release of CD34+ from bone marrow into peripheral circulation (33), it will be important to investigate the relation between miR-126-3p and hematopoietic precursor cells in NTZ-treated patients with MS.

MicroRNA-126-3p serum concentration in MS subjects before treatment showed a significant inverse correlation with disease duration and with age, suggesting that the age and time from the MS onset are both factors contributing to its expression. The negative correlation observed between miR-126-3p concentration and age in patients with MS is in contrast with the age-related miR-126-3p plasma concentration increases described in healthy individuals (34). Thus, miR-126-3p synthesis and release by extracellular vesicles from ECs was shown to be increased in senescence, and miR126-3p is generally considered an “age associated biomarker.” As miR-126-3p downregulates the activity of SPRED1, an inhibitor of the Ras/MAPK signaling pathway, this was suggested to be a protective mechanism that contrasts cellular senescence (35). Moreover, miR-126 can regulate vascular inflammation, inhibiting the expression of VCAM-1 in ECs, and limiting the recruitment of inflammatory cells (30). These protective mechanisms are impaired in diseases, such as diabetes: in this situation hyperglycemia reduces miR-126-3p concentration in plasma contributing to the pathology. Our results suggest that the pro-inflammatory milieu characteristic of MS could have an impact on circulatory mir-126-3p, leading to its downregulation or, alternatively, that an initial reduction in mir-126-3p is a factor in favoring the generation of such milieu. The complex interaction between inflammation and miR-126-3p in autoimmune diseases, such as MS, is further reinforced by results showing that the increased concentration of TNF-alpha and IFN-gamma seen in this disease leads to a reduction of mir-126-3p in brain ECs, resulting in an increased VCAM-1 expression and augmented leukocytes adhesion to the brain endothelium (36).

Serum concentration of sVCAM-1, a molecular target of mir-126-3p was not different at baseline in RRMS compared with HC. This result confirms most (37, 38) but not all previously published data (39, 40); the heterogeneity of patients with MS, different timing of sampling in the relation of activity of the disease and the use of diverse methods could influence these discrepancies. After therapy initiation, sVCAM-1 serum concentration decreased concurrently with that of miR-126-3p. Of note, no differences could be detected in sVCAM-1 serum concentration either in the patient who developed PML or when patients were subdivided based on the reactivation/lack of JCPyV replication, suggesting that the mir-126-3p/sVCAM-1 interaction does not play a role in this phenomenon.

In contrast with these findings, *Spi-B* and *POU2AF1* gene expression was significantly increased at baseline in RRMS PBMC compared with HC. miR-126-3p regulates POU2AF1expression; this transcription factor modulates the expression of *Spi-B*, a molecule that ligates the JCPyV promoter (41). POU2AF1 is constitutively expressed in B lymphocytes and regulates the function and differentiation of these cells. As a consequence, changes in miR-126-3p concentration could activate B lymphocytes and directly stimulate JCPyV replication. The transcription of these transcription factors was upregulated by NTZ; these results are in line with previous data.^8^ Notably, the effect of treatment on *Spi-B* and *POU2AF1* expression was significantly more evident in patients in whom NTZ was associated with increased asymptomatic viral reactivation and augmented miR-126-3p serum concentration. These results support the hypothesis that Spi-B and POU2AF1 upregulation during treatment could facilitate JCPyV reactivation in PBMCs, and in the presence of other concomitant event (sequence mutation in viral regulatory region, migration in CNS, and immunosuppression) could play an important role in the PML development.

Importantly, the results suggest that the presence of disease activity (relapses, MRI activity, or EDSS progression) during NTZ did not influence miR-126-3p, *SpiB*, and *POU2AF1* expression, but more observations will be needed to confirm these findings.

The increased expression of miR-126-3p observed in relation with JCPyV activity is in accordance with results obtained in the only patients with RRMS included in the study who developed PML. In this patient, miR-126-3p serum concentration was greatly augmented at every time point. PBMCs were not available from this patient; previous data, nevertheless, showed that Spi-B and POU2AF1 expression is greatly increased in patients with MS who develop PML upon NTZ therapy (7). Taken together, these results lend further support to the hypothesis that NTZ-associated JCPyV reactivation is strongly correlated with the ability of NTZ to modulate the miR-126-3p/*POU2AF1/Spi-B* axis.

The clear limitation of this study is the limited number of analyzed individuals. Moreover, the findings observed in the subject with PML need to be confirmed in more patients that developed PML after NTZ, or as a consequence of the use of other disease modifying therapies. This caveat notwithstanding, our results might offer an explanation to the reactivation of JCPyV replication that can be observed in NTZ-treated patients with MS, and suggest that miR-126-3p, *POU2AF1*, and *Spi-B* could be useful biomarkers to monitor JCPyV reactivation. Additional studies in the bigger cohort of patients will be needed to validate these data, as well as to analyze the possible impact of other disease modifying treatments on these molecular parameters.

## Data Availability Statement

The original contributions presented in the study are included in the article/Supplementary Material, further inquiries can be directed to the corresponding author.

## Ethics Statement

The studies involving human participants were reviewed and approved by Ethics Committee of Don Gnocchi Foundation IRCCS. The patients/participants provided their written informed consent to participate in this study.

## Author Contributions

RM and MC designed the experiments. DC, DG, and ES enrolled the subjects and collected the biological samples. SA and AH performed the experiments and collect the data. RM and SA analyzed the data. RM, SA, and MC interpreted the data and drafted the manuscript. All authors approved the final version.

## Funding

This study was funded by the 2019–2021 Ricerca Corrente, Italian Ministry of Health.

## Conflict of Interest

The authors declare that the research was conducted in the absence of any commercial or financial relationships that could be construed as a potential conflict of interest.

## Publisher's Note

All claims expressed in this article are solely those of the authors and do not necessarily represent those of their affiliated organizations, or those of the publisher, the editors and the reviewers. Any product that may be evaluated in this article, or claim that may be made by its manufacturer, is not guaranteed or endorsed by the publisher.
