# Supplementary material for: Alterations of the miR-126-3p/POU2AF1/Spi-B Axis and JCPyV Reactivation in Multiple Sclerosis Patients Receiving Natalizumab
Source: Front Neurol. 2022 Mar 11;13:819911. doi: 10.3389/fneur.2022.819911 (PMC8963350; doi:10.3389/fneur.2022.819911)
Supplement: Supplementary file 1 [file Table_1.DOCX]

Supplementary Material

**Supplementary Figure 1.** JCPyV detection in Multiple Sclerosis (MS) patients before and during 24-month therapy with Natalizumab (NTZ). Information about JCPyV infection and activity as well as presence of antibodies (Ab), DNA detection in urine (U) serum (S) or blood were shown. Black boxes correspond to JCPyV DNA positive samples, gray boxes to negative samples. na: not available; nd: not detected; Mo: month.
